# Louisville Dental Association

**Published:** 1865

**Authors:** Chas. E. Dunn

**Affiliations:** Louisville, Ky.


*Louisville, Ky., March 15th*, 1865.

Editors Register: At the last regular meeting of the Louisville Dental Association, the following preamble and resolution was adopted; and additional, that you be requested to publish the same. Chas. E. Dunn, Cor. Sec.

Whereas, a difference of opinion exists among the members of the Louisville Dental Association as to the utility of lancing the gums preparatory to extracting teeth, and also as to the use of water after the operation, therefore,

*Resolved*, That dental societies and practioners in other cities and localities be requested to report their practice in these particulars, and societies their debates on the subjects, through the *Cosmos*, *Register* or other dental journals, or to this society direct.

